# *Solanum melongena* L. Extract Protects Retinal Pigment Epithelial Cells from Blue Light-Induced Phototoxicity in In Vitro and In Vivo Models

**DOI:** 10.3390/nu13020359

**Published:** 2021-01-25

**Authors:** Thu Nguyen Minh Pham, Chae-Young Shin, Seo Hyun Park, Taek Hwan Lee, Hyeon Yeol Ryu, Sung-Bae Kim, Kwang Auh, Kwang Won Jeong

**Affiliations:** 1 Gachon Research Institute of Pharmaceutical Sciences, College of Pharmacy, Gachon University, Incheon 21936, Korea; phamnguyenminhthud12@gmail.com (T.N.M.P.); codud9921@naver.com (C.-Y.S.); 2 Department of Pharmacognosy, Faculty of Pharmacy, Hong Bang International University, Ho Chi Minh 215, Vietnam; 3 R&D Center, Ahn-Gook Health Co., Ltd., Seoul 06164, Korea; shpark@ag-health.com (S.H.P.); lth0717@naver.com (T.H.L.); khan@ag-health.com (K.A.); 4 Korea Conformity Laboratories, Incheon 21999, Korea; rhyckato98@kcl.re.kr (H.Y.R.); suaa10@kcl.re.kr (S.-B.K.)

**Keywords:** eggplant extract, blue light, A2E, age-related macular degeneration, chlorogenic acid

## Abstract

N-retinylidene-N-retinylethanolamine (A2E) accumulation in the retina is a prominent marker of retinal degenerative diseases. Blue light exposure is considered as an important factor contributing to dry age-related macular degeneration (AMD). Eggplant and its constituents have been shown to confer health benefits, but their therapeutic effects on dry AMD remain incompletely understood. In this study, we showed that an extract of *Solanum melongena* L. (EPX) protected A2E-laden ARPE-19 cells against blue light-induced cell death via attenuating reactive oxygen species. Transcriptomic analysis demonstrated that blue light modulated the expression of genes associated with stress response, inflammation, and cell death, and EPX suppressed the inflammatory pathway induced by blue light in A2E-laden ARPE-19 cells by inhibiting the nuclear translocation of nuclear factor kappa B and transcription of pro-inflammatory genes (*CXCL8* and *IL1B*). The degradation of intracellular A2E was considered the major mechanism underlying the protective effect of EPX. Moreover, chlorogenic acid isolated from EPX exerted protective effects against blue light-induced cell damage in A2E-laden ARPE-19 cells. In vivo, EPX administration in BALB/c mice reduced the fundus damage and degeneration of the retinal layer in a blue light-induced retinal damage model. Collectively, our findings suggest the potential role of *Solanum melongena* L. extract for AMD treatment.

## 1. Introduction

Age-related macular degeneration (AMD) is a multifactorial disease that damages the macular area, leading to the loss of central vision by modulating different physiological pathways. Apart from the genetic causes, smoking and exposure to sunlight are the other risk factors associated with macular degeneration [1]. Early-stage AMD is characterized by the accumulation of lipofuscin in the retinal pigment epithelium (RPE) and deposition of drusen (yellowish-white deposits) in the Bruch’s membrane. The late-stage AMD comprises two distinct types, dry AMD with atrophy of RPE cells and wet AMD with choroidal neovascularization. AMD is one of the three causes of vision loss worldwide [2]. At present, anti-vascular endothelial growth factor (VEGF) agents form the primary treatment modality for wet AMD and are shown to significantly improve central vision loss [3]. However, evidence-based treatment for dry AMD remains unavailable, and research is underway to identify novel therapeutic agents [1,4]. Several clinical studies in patients with moderate or severe AMD using antioxidant supplements (AREDS studies) have concluded with unsatisfactory results. Therefore, the development of treating dry AMD is imperative and efforts have been directed to develop treatment strategies using natural extracts [4,5,6].

Aging is associated with the accumulation of lipofuscin in the retina, including the RPE cells. Bis-retinoid N-retinylidene-N-retinylethanolamine (A2E), produced from the reaction between vitamin A and phosphatidylethanolamine during the visual cycle, is an important component of lipofuscin [7]. A2E is known to be associated with atrophic AMD owing to its structural properties [8]. In vitro, A2E is cleaved by blue light to produce reactive oxygen species (ROS) and other by-products [9,10]. A2E-accumulated RPE cells respond to A2E photo-oxidation by triggering the activation of the complement system and consequently inducing chronic inflammation [11]. Further, A2E activated by low-wavelength light directly destroys the DNA of RPE cells, thereby increasing the cellular damage [12]. It was recently shown that photo-oxidation of A2E induced by blue light results in lysosomal damage in retinal cells and disrupts the intracellular autophagy process [13]. Therefore, reduction in oxidative stress, inflammatory signaling, and reactivation of autophagy are considered important approaches to block the phototoxic effect of A2E in AMD pathology [3,14,15,16].

Eggplant, an edible plant rich in beneficial nutrients such as vitamins, phenols, and antioxidants, has been extensively investigated for its antioxidant properties [17,18,19]. In particular, *Solanum melongena* L. contains high levels of components with strong radical-scavenging activities [19,20]. In RAW264.7 cells, eggplant extracts reduced inflammation by scavenging free radicals and reducing the production of nitric oxide, pro-inflammatory cytokines, and prostaglandin E2 [21,22]. In a mouse paw edema model, aqueous extract of *Solanum melongena* inhibited myeloperoxidase activity and tumor necrosis factor-alpha (TNFα) expression induced by proteinase-activated receptor 2 (PAR2) agonists [23].

Despite these benefits, the protective effects of *Solanum melongena* in dry AMD remain unclear. Therefore, we investigated the protective action of *Solanum melongena* extracts (EPX) on phototoxicity in a blue light-induced retinal damage model. Using in vitro and in vivo models, we showed that EPX effectively inhibited blue light-induced macular degeneration. Further, we investigated whether the antioxidant effect of EPX alleviated inflammatory processes and apoptosis related to blue light-induced retinal cell damage and studied the underlying mechanism by evaluating gene expression changes by RNA sequencing. In addition, we investigated whether EPX removes accumulated A2E in retinal pigment epithelial cells. Our results suggest the potential application of EPX for the treatment of dry AMD.

## 2. Materials and Methods

### 2.1. Cell Culture

Human retinal pigment epithelial cells (ARPE-19), purchased from the American Type Culture Collection (Manassas, VA, USA), were maintained in Dulbecco’s Modified Eagle’s medium F-12 (DMEM/F-12; WELGENE, Gyeongsan, Korea) supplemented with 10% fetal bovine serum (Thermo Fisher, Waltham, MA, USA), 100 U/mL penicillin, and 100 μg/mL streptomycin (Thermo Fisher) at 37 °C in a humidified atmosphere containing 5% CO_2_.

### 2.2. Preparation of EPX

Eggplant (*Solanum melongena* L.) was obtained from Shinwoo Co., Ltd. (Anyang-si, Gyeonggi-do, Korea). Air-dried eggplants were extracted with a 50% ethanol for 24 h at room temperature and the obtained extract was filtered. After filtration, the ethanolic extract was evaporated under reduced pressure using a rotary vacuum evaporator (EYELA, Tokyo, Japan). The concentrated extract was powdered and used for analyses. The EPX was dissolved in dimethyl sulfoxide (DMSO) and filtered through a 0.2 μm PTFE syringe filter (Corning Inc., Corning, NY, USA) for in vitro experiments.

### 2.3. High-Performance Liquid Chromatography (HPLC)

Quantitative analysis of bioactive polyphenolic compounds in EPX was performed using a Waters e2695 separation module and a 2998 photodiode array detector (Waters Co., Milford, MA, USA). EPX was separated on a Phenomenex Luna C 18(2) column (250 × 4.6 mm, 5 μm, Phenomenex Co., Torrance, CA, USA). The mobile phases comprised 0.1% (*v*/*v*) trifluoroacetic acid in water (solvent A) and acetonitrile (solvent B). The gradient elution conditions used were as follows: 0–10 min, 10% B; 10–60 min, 10–60% B; 60–61 min, 60–100% B; 61–71 min, 100% B; 71–72 min, 100–10% B; 72–90 min, 10% B. The column temperature was maintained at 40°C, the sample injection volume was 10 μL, and the flow rate was 1 mL/min. All samples were filtered through a 0.4 μm syringe filter prior to injection.

### 2.4. A2E-Blue Light ARPE-19 Cell Model and Cell Viability Assay

ARPE-19 cells (10^4^ cells/well) were seeded in 6-well plates and treated three times with 20 μM A2E (AptaBio, Yongin, Korea) over a period of 6 days at 48 h intervals. Then, the cells were treated twice with EPX for two consecutive days, illuminated by blue light (430 nm, 8000 lux) for 30 min, and incubated for an additional 24 h [24]. EZ-Cytox (DoGen bio, Seoul, Korea) was used to monitor cell viability. Briefly, cells were seeded (5 × 10^3^ cells/well) in a 96-well plate and treated with EPX (50–500 μg/mL) for 24 h. Then, 10 μL of EZ-Cytox was added into each well. EZ-Cytox is a water-soluble tetrazolium salt that forms water-soluble formazan by cellular dehydrogenase. The absorbance was measured at 450 nm using a BioTeK microplate reader (Winooski, VT, USA).

### 2.5. ROS Assay

ROS quantitation was performed based on a previously published method [13]. Briefly, ARPE-19 cells were treated with 10 μM 2,7-dichlorodihydrofluorescein diacetate (DCFH-DA; Sigma-Aldrich, MO, USA) in serum-free media and incubated at 37°C for 10 min. The level of ROS in cells was monitored using an inverted fluorescence microscope (Nikon eclipse Ti-U, Nikon Instruments Inc., Tokyo, Japan). The cells were lysed using radioimmunoprecipitation assay (RIPA) cell lysis buffer (50 mM Tris-HCl pH 8.0, 150 mM sodium chloride [NaCl], 2 mM ethylenediaminetetraacetic acid [EDTA], 1% sodium dodecyl sulfate [SDS], 1% sodium deoxycholate, and 1% NP-40), and the lysate was transferred into a 96-well white plate. The fluorescence intensity was measured using Multimode Plate Reader Victor X3 (Perkin Elmer, Waltham, MA, USA) at excitation and emission wavelengths of 480 and 530 nm, respectively.

### 2.6. Western Immunoblotting

Western immunoblotting was performed as previously described [25]. Briefly, cell lysates were prepared using RIPA buffer (50 mM Tris-HCl pH 8.0, 150 mM NaCl, 2 mM EDTA, 1% SDS, 1% sodium deoxycholate, and 1% NP-40). The following primary antibodies were used for immunoblotting: anti-Poly (ADP-ribose) polymerase (PARP; Cell Signaling, MA, USA), anti-p65 (Cell Signaling), anti-histone H3 (Abcam, Cambridge, UK), anti-α-tubulin (Santa Cruz, TX, USA), and anti-β-actin (Santa Cruz). Quantitative intensities were evaluated using Image Lab 5.1 by normalization to the β-actin level.

### 2.7. RNA Sequencing and Pathway Analysis

RNA sequencing was performed as previously described [26]. Briefly, total RNA was isolated 24 h after exposing cells to blue light, using TRIzol RNA Isolation reagent (Invitrogen, Carlsbad, CA, USA), and genomic DNA was removed using an RNeasy Mini Kit (QIAGEN, Hilden, Germany). Purified RNA was processed to prepare an mRNA-seq library using the TruSeq Stranded mRNA Kit (Illumina, San Diego, CA, USA). Each produced library was sequenced using the Illumina NextSeq instrument (Illumina). The raw data were converted into sequence data and stored in FASTQ format. Gene set enrichment analysis (GSEA, www.broadinginstitute.org/gsea) was performed to investigate the differential biological effects on the A2E-blue light ARPE-19 cell model [27,28] in the presence and absence of EPX. Genes showing an absolute fold-change of at least 2 at a false discovery rate (FDR) < 0.05 were considered differentially expressed.

### 2.8. Reverse-Transcriptase Quantitative Polymerase Chain Reaction (RT-qPCR)

The mRNA was reverse-transcribed using the iScript cDNA Synthesis Kit (Bio-Rad Laboratories, Hercules, CA, USA) at a total volume of 20 μL. RT-qPCR was performed on a Roche LightCycler^®^480II system using SYBR Green I Master (Roche, South San Francisco, CA, USA). The primers used in RT-qPCR are listed in Supplementary Table S1. mRNA expression levels were normalized to 18S rRNA levels.

### 2.9. Isolation of Nuclear or Cytoplasmic Proteins

Cytoplasmic proteins were extracted using a cytoplasmic extract buffer (10 mM HEPES, 60 mM potassium chloride (KCl), 1 mM EDTA, 0.03% NP-40, and 1 mM dithiothreitol with protease inhibitor). The nuclei obtained from centrifugation were further lysed using a nuclear extract buffer (20 mM Tris-HCl, 420 mM NaCl, 1.5 mM magnesium chloride (MgCl_2_), 0.2 mM EDTA, and 25% glycerol with protease inhibitor) to obtain nuclear proteins.

### 2.10. A2E Assay

The A2E assay was performed using A2E-BDP as previously described [24]. ARPE-19 cells (5 × 10^3^ cells/well) were seeded in a 96-well white tissue culture plate. For the A2E accumulation assay, cells pretreated with EPX for 24 h were incubated with 10 μM A2E-BDP for 24 h. For the A2E degradation assay, A2E-laden cells were treated with EPX for 24 h. Fluorescence intensity was visualized using an inverted fluorescence microscope (Nikon eclipse Ti-U) and measured using a fluorescent microplate reader (Victor X3).

### 2.11. Animals and Blue Light Exposure

Five-week-old male BALB/c mice were purchased from Orient Bio Inc. (Seongnam, Korea) and housed in individual polycarbonate cages under specific pathogen-free conditions. The animals were acclimated to the environment for a week prior to the experiment. Experimental procedures were approved by the Institutional Animal Care and Use Committee of Korea Conformity Laboratories (approval no. IA19-02639) and performed in accordance with the relevant guidelines. The mice were randomly divided into five groups (*n* = 10 each) as follows: NC group (normal control, treated with vehicle), blue light (BL) group (negative control, treated with vehicle and blue light), EPX 100 group (EPX 100 mg/kg each day + blue light), EPX 200 group (EPX 200 mg/kg each day + blue light), and lutein group (positive control, treated with lutein 40 mg/kg each day + blue light). The mice were provided a standard laboratory diet (Teklad Certified Irradiated Global 18% Protein Rodent Diet, ENVIGO) and purified water ad libitum during the experimental period and weighed once a week after study initiation.

**Figure 1:**
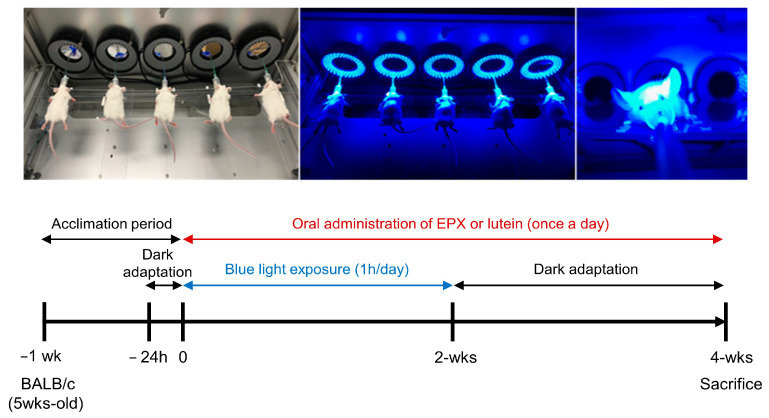
Schematic representation of the in vivo experimental design. Blue light exposure was performed 14 times (1 h/day) at 10,000 lux using a blue light irradiator after anesthetizing mice with isoflurane.

The schedule of BL exposure and each treatment is shown schematically in Figure 1. After adaptation to dark for 24 h, animals were exposed to BL and treated with different samples for 4 weeks. The mice were orally administered EPX or lutein (10% FloraGlo lutein powder, Aurobindo Pharma Ltd.) once a day before BL exposure. EPX and lutein were dissolved in sterile distilled water (used as a vehicle). After the last irradiation with BL, the mice were maintained in darkness for 2 weeks and then euthanized for eye excision.

### 2.12. Hematoxylin and Eosin (H&E) Staining

After euthanasia, both ocular tissues were immediately excised and fixed in Davidson’s solution. H&E staining was performed, and the average value of outer nuclear layer (ONL) thickness was measured at 140 μm (left/right) from the retinal nerve center using the Axio Vision SE644 (ZEISS) program. After measuring the thickness of ONL in the opposite eyeball in a similar manner, the average value obtained from both eyes was used as the final ONL thickness. Similarly, the number of RPE cells was determined by counting the nuclei in a 200 μm wide region of section located at equal distance from the retinal nerve center. Average cell numbers were analyzed using ImageJ (National Institutes of Health, Bethesda, MD, USA).

### 2.13. Fundus Imaging

Both eyes of all mice were examined once a week before initiating the treatment. After dilating the pupil by instilling a mydriatic drug, the fundus area of the eye was observed using a fundus camera (Genesis-D, Kowa Co. Ltd., Japan). Images were obtained for each mouse at the end of BL exposure and test substance treatment periods.

### 2.14. Statistical Analysis

For the in vitro study, statistical analysis was performed using GraphPad Prism 5.01 (GraphPad, San Diego, CA, USA). Data are presented as means ± standard deviation (S.D.). Significant differences between the compared groups were calculated by paired *t*-test. Results with *p* < 0.05 were considered statistically significant. For the in vivo study, statistical analysis was performed using the SPSS 12.0 K program (SPSS, Chicago, IL, USA). Data are presented as the mean ± S.D. from independent experiments. Significant differences among groups were analyzed using the Student’s *t*-test or one-way analysis of variance (ANOVA) followed by Duncan’s test for homogeneity of variance or Dunnett’s T3 test for heterogeneity of variance. Results with *p* < 0.05 were considered statistically significant.

## 3. Results

### 3.1. Protection of Retinal Cells by EPX

**Figure 2:**
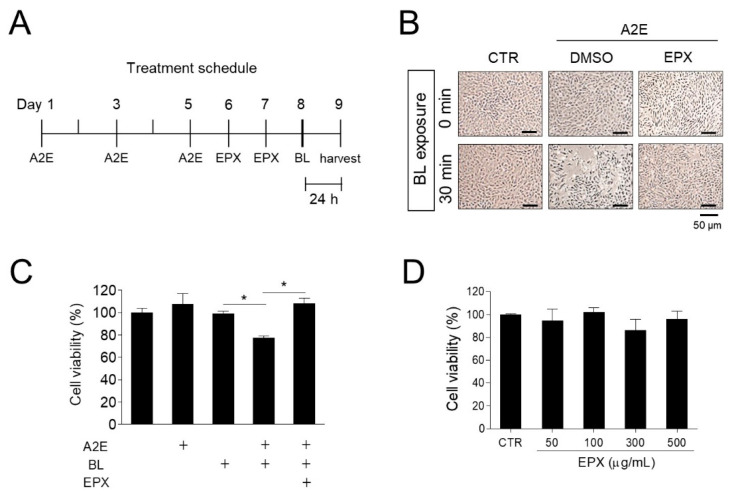
Protective effect of EPX on phototoxicity induced by blue light in A2E-laden ARPE-19 cells. (**A**) Treatment schedule of A2E and EPX. A2E—Bis-retinoid N-retinylidene-N-retinylethanolamine; EPX—eggplant extract; BL—blue light. (**B**,**C**) Protection of retinal cells by EPX. ARPE-19 cells were treated with fresh A2E (20 μM) every 2 days for a period of 6 days and then exposed twice to vehicle (DMSO) or EPX (25 μg/mL) for 48 h. Next, the cells were exposed to blue light (BL, 430 nm, 8000 lux) for 30 min. After incubation for 24 h, cell viability was visualized using an inverted phase-contrast microscope (B) and measured using EZ-Cytox (C). CTR—control; DMSO—dimethyl sulfoxide; +—sample treatment. The results are presented as the mean ± S.D. (*n* = 3); * *p* < 0.05. (**D**) Viability of ARPE-19 cells treated with EPX (50 to 500 μg/mL) for 24 h.

First, we investigated the protective effect of EPX in an in vitro A2E-blue light-induced phototoxicity model established using human retinal pigment epithelial cells. Phototoxicity was induced by exposing A2E-laden ARPE-19 cells to BL (430 nm) for 30 min. Cells were treated twice with EPX (20 μg/mL) for 2 days before BL irradiation (Figure 2A). Compared to the control group, A2E treatment or BL exposure alone did not affect the viability of ARPE-19 cells; however, the viability of A2E-laden ARPE-19 cells significantly decreased (−26.63%) upon irradiation with BL. On the contrary, EPX treatment significantly inhibited the decrease in the viability of A2E-laden ARPE-19 cells irradiated with BL (Figure 2B,C). In the presence of A2E, EPX treatment without BL did not affect the cell viability. The EPX used in the experiment showed no significant cytotoxicity against ARPE-19 cells at concentrations up to 500 μg/mL (Figure 2D). These data suggest that EPX could protect A2E-loaded ARPE-19 cells from blue light-induced damage.

### 3.2. Decrease in ROS Production in RPE Cells by EPX

**Figure 3:**
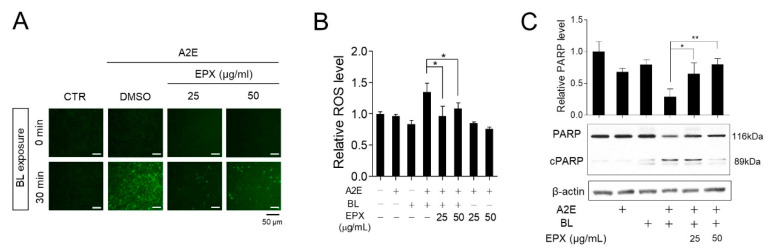
EPX decreases ROS production and apoptosis induced by blue light in A2E-laden retinal pigment epithelium (RPE) cells. (**A**,**B**) Decrease in ROS production by EPX. After treatment with EPX (25–50 μg/mL), ROS level was monitored by DCFH-DA (10 μM, 10 min) in blue light (BL)-exposed A2E-laden RPE cells. The fluorescence was visualized by fluorescence microscopy (**A**) and quantified using a fluorescence microplate reader (**B**). CTR—control; DMSO—dimethyl sulfoxide; BL—blue light; + —sample treatment; ROS—reactive oxygen species. The results are presented as the mean ± S.D. (*n* = 4); * *p* < 0.05. (**C**) Reduction of PARP cleavage by EPX. A2E-laden RPE cells were treated with EPX (25–50 μg/mL) for 48 h before BL illumination. Western immunoblot analysis indicated full-length PARP and cleaved PARP (cPARP) protein expression, and the band intensities were quantified by densitometric analysis. cPARP—cleaved PARP. The results are presented as mean ± S.D. (*n* = 3); * *p* < 0.05; ** *p* < 0.01.

Dry AMD is associated with the appearance of lipofuscin (drusen) in the retina [29,30]. Drusen progression, including A2E synthesis and lipofuscin granulation, is a characteristic feature of dry AMD. ROS is produced during the photo-oxidation of A2E by BL [31,32]. BL in the visible region is also a risk factor for AMD [33,34,35,36], consistent with the induction of ROS accumulation and death in cells loaded with A2E [37,38]. Eggplant is known to contain several antioxidants [39]. Therefore, we hypothesized that the inhibition of ROS production induced by A2E and BL might be one of the mechanisms underlying the protective effects of EPX. Cells treated with A2E and BL showed a significant increase in ROS levels than ARPE-19 cells that received either A2E or BL. Treatment with EPX significantly reduced the A2E + BL-induced intracellular ROS production (Figure 3A,B). BL irradiation was shown to induce apoptosis of A2E-treated RPE cells [40,41]. We evaluated the effect of EPX on PARP activation (PARP cleavage) as an apoptosis marker in the A2E-BL model. After BL irradiation, RPE cells containing A2E showed reduced PARP level. In contrast, PARP in EPX-treated cells was maintained at the control level (Figure 3C). These observations suggest that the inhibition of ROS production induced by BL in A2E-laden RPE cells is one of the protective mechanisms of EPX.

### 3.3. Effect of EPX on Global Gene Expression in ARPE-19 Cells

**Figure 4:**
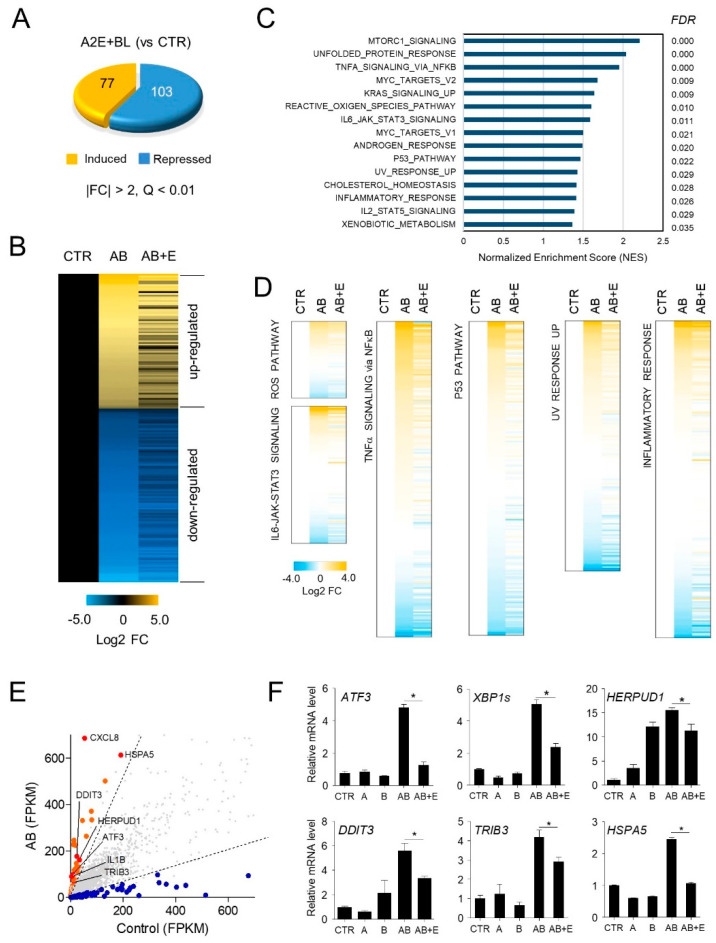
Effect of EPX on genome-wide gene expression in ARPE-19 cells. (**A**) Differentially expressed genes by A2E and BL in ARPE-19 cells. A2E-laden ARPE-19 cells were exposed to blue light (BL), as shown in Figure 2A. RNA-seq analysis showed differentially expressed genes (DEGs) after BL exposure in A2E-laden RPE cells (A2E + BL) compared to untreated cells (CTR). FC—fold change; A2E—Bis-retinoid N-retinylidene-N-retinylethanolamine; BL—blue light. (**B**) Heatmap generated from three groups, including A2E-free ARPE-19 cells (CTR) and BL-stimulated A2E-laden RPE cells, in the absence (AB) and presence of EPX (AB + E). Values represent the log2 fold-change (FDR < 0.01) relative to CTR. (**C**–**E**) Pathways significantly altered after exposure to A2E and BL. From GSEA according to Hallmark collection, statistical significance in molecular functions (NES > 1, FDR < 0.05) is listed. AB—A2E+blue light; E—eggplant extract. (**F**) Validation of DEGs by RT-qPCR. *ATF3*—activating transcription factor 3; *XBP1s*—spliced X-box binding protein 1; *TRIB3*—tribbles pseudokinase 3; *DDIT3*—DNA damage-inducible transcript 3; *HERPUD1*—homocysteine-inducible endoplasmic reticulum protein with ubiquitin-like domain 1; *HSPA5*—heat shock protein family A (Hsp70) member 5. The results are presented as the mean ± S.D. (*n* = 4); * *p* < 0.05.

To further understand the mechanism underlying the action of EPX, we performed RNA-seq to examine the effect of EPX on gene expression. Among the 11,895 genes expressed in ARPE-19 cells, those showing significant changes in expression (|FC| > 2 and FDR < 0.01) after treatment with A2E and BL irradiation were identified. Hence, a total of 180 genes (77 upregulated and 103 downregulated genes) were differentially expressed in ARPE-19 cells treated with A2E and BL compared to untreated controls (Figure 4A). The expression of most of the genes altered following A2E and blue light exposure, was restored to the levels observed in control cells following treatment with EPX (Figure 4B). Pathway analysis showed that the genes involved in pathways related to inflammation and apoptosis, such as TNFα signaling via nuclear factor kappa B (NF-κB; FDR = 0.000), ROS pathway (FDR = 0.010), interleukin 6 (IL6) Janus kinase (JAK) signal transducer and activator of transcription 3 (STAT3) signaling (FDR = 0.011), p53 pathway (FDR = 0.022), UV response upregulated (FDR = 0.029), and inflammatory response (FDR = 0.026) were significantly altered after exposure to A2E and BL (Figure 4C). The expression of these dysregulated genes was restored to the control levels after EPX treatment (Figure 4D). The expression of the genes involved in NF-κB pathway was further verified using RT-qPCR (Figure 4E,F). These results suggest that EPX has a potential role in regulating the upstream process related to the AMD pathogenesis.

### 3.4. EPX Inhibits the NF-κB Pathway Activated by BL in RPE Cells

**Figure 5:**
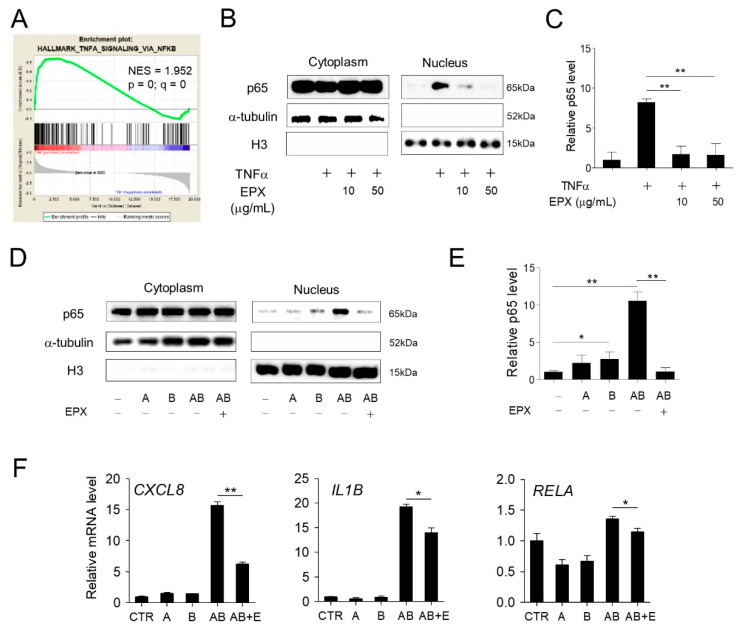
Eggplant extract inhibits the NF-κB pathway activated by blue light (BL) in RPE cells. (**A**) GSEA showing that the genes modulated by BL and A2E were significantly enriched in the NF-κB pathway. NES—normalized enrichment score. (**B**) Inhibition of NF-κB signaling by EPX. EPX inhibited the nuclear translocation of p65 induced by TNFα in ARPE-19 cells. The p65 protein level in the cytoplasm or nuclear fraction was determined by western immunoblot using a specific anti-p65 antibody. α-tubulin and histone H3 were used as internal controls for cytoplasmic and nuclear fractions, respectively. H3—histone H3; TNF—tumor necrosis factor; EPX—eggplant extract; + —sample treatment. (**C**) Quantification of p65 level in the nucleus. The nuclear p65 level was quantified using ImageJ. The nuclear protein level of p65 was normalized to histone H3 level. The results are presented as the mean ± S.D. (*n* = 3) (**D**,**E**) The effect of EPX on A2E+BL-induced NF-κB pathway. EPX (25 μg/mL) inhibited the translocation of p65 induced by BL in A2E-laden ARPE-19 cells. p65 protein level was determined and quantified, as illustrated in Figure 5B,C. A—A2E; B—blue light; AB—A2E+blue light. (**F**) The effect of EPX (25 μg/mL) on the expression of pro-inflammatory genes determined by RT-qPCR. The mRNA levels were normalized to *18S* rRNA level. *CXCL8*—C-X-C motif chemokine ligand 8; *IL1B*—interleukin 1 beta; *RELA*—proto-oncogene, NF-κB subunit. E—eggplant extract. The results are presented as the mean ± S.D. (*n* = 3); * *p* < 0.05; ** *p* < 0.01.

The activation of NF-κB-mediated inflammatory pathways by BL irradiation in human retinal cells has been previously reported [25]. GSEA [28] using RNA-seq results also revealed the enrichment of genes involved in TNFα signaling via the NF-κB pathway after exposure to A2E and BL (Figure 5A). In addition, we previously confirmed that EPX treatment inhibited the change in the expression of NF-κB target genes (Figure 4F). To determine whether EPX affects the NF-κB activation induced by TNFα, ARPE-19 cells were treated with TNFα, and the change in the nuclear translocation of p65 protein was evaluated. The nuclear p65 protein levels increased after TNFα treatment, but this upregulation significantly decreased after EPX treatment (Figure 5B,C). Moreover, the p65 protein level in the nucleus increased in response to A2E + BL, but this effect was markedly decreased by EPX treatment (Figure 5D,E). Further, the increase in the expression of endogenous NF-κB target genes by A2E and BL irradiation was significantly reduced by EPX treatment (Figure 5F). These results suggest that EPX exerts a cytoprotective effect in RPE cells by inhibiting NF-κB activation induced by A2E and BL.

### 3.5. Removal of Intracellular A2E by EPX

**Figure 6:**
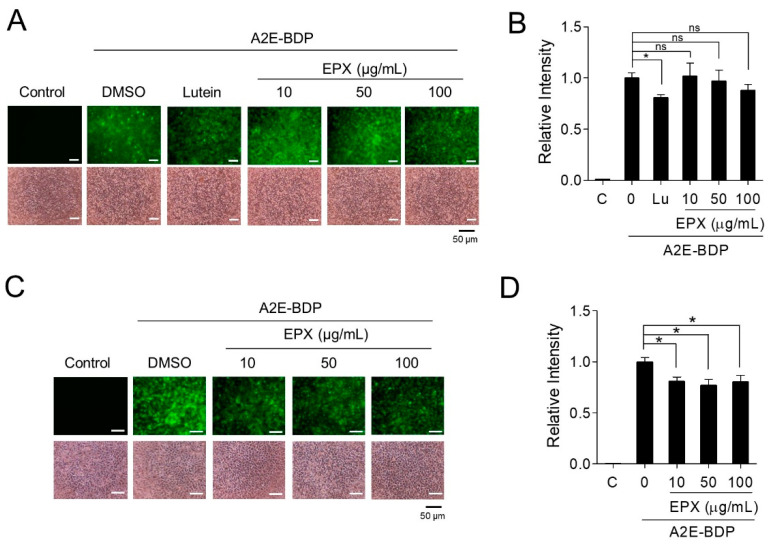
EPX decreases the intracellular A2E level. (**A**,**B**) The effect of EPX on A2E accumulation in ARPE-19 cells. ARPE-19 cells were treated with EPX (10–100 μg/mL) for 24 h before exposure to A2E-BDP (10 μM). The fluorescence was visualized using a fluorescence microscope (**A**, upper panels). Lutein (30 μM) was used as a positive control. Cell viability was determined using a phase-contrast microscope (**A**, bottom panels). The relative fluorescence intensity was measured on a fluorescence microplate reader. The results are presented as the mean ± S.D. (*n* = 3). Lutein (Lu) was used as a positive control. EPX—eggplant extract; ns—not significant. (**C**,**D**) The effect of EPX on the degradation of A2E in ARPE-19 cells. Cells were incubated with A2E-BDP (10 μM) before treatment with EPX (10–100 μg/mL). Intracellular A2E-BDP level was monitored, as shown in Figure 6A,B. The results are presented as the mean ± S.D. (*n* = 4); * *p* < 0.05.

To investigate the detailed mechanism underlying the protective effect of EPX on retinal cells, we used fluorescence-based A2E (A2E-BDP) to assess the amount of A2E in the cells. A2E-BDP is a fluorescently-labeled A2E used to track the localization of A2E into organelles within cells [24]. The effects of EPX on A2E were evaluated in two ways. First, to examine the effect of EPX on the accumulation of A2E, ARPE-19 cells were pretreated with EPX for 24 h before A2E treatment. The accumulated A2E-BDP in ARPE-19 cells was monitored by fluorescence microscopy and fluorescence spectrophotometry. EPX treatment in ARPE-19 cells did not affect the intracellular accumulation of A2E (Figure 6A,B). Lutein, used as a positive control, significantly inhibited A2E accumulation [42]. Next, the effect of EPX on the degradation of A2E in RPE cells was examined. After treating A2E-BDP-laden ARPE-19 cells with EPX for 24 h, the remaining A2E-BDP was measured. EPX significantly decreased the intracellular A2E-BDP level in cells even at the lowest concentration (10 μg/mL) without affecting cell viability (Figure 6C,D). These results indicate that the main mechanism by which EPX protects retinal cells is via degrading the intracellular A2E. Furthermore, these results explain how EPX inhibits all the downstream effects (e.g., ROS generation, inflammation, and apoptosis) induced by A2E photo-oxidation by BL.

### 3.6. Chlorogenic Acid (CGA) Is an Active Substance in EPX that Protects Retinal Cells

**Figure 7:**
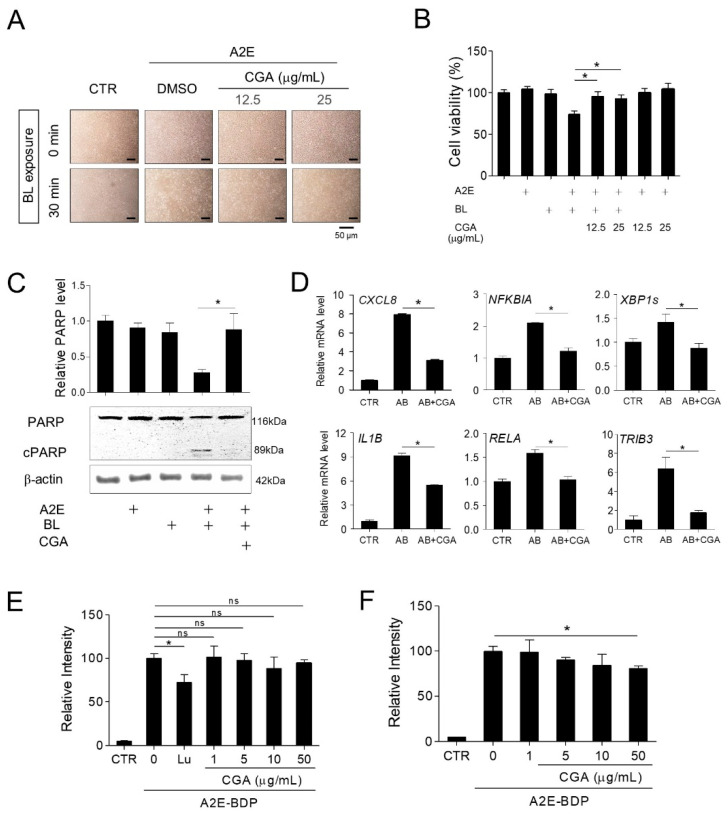
Chlorogenic acid (CGA) is an active substance in EPX that protects retinal cells from A2E- and blue light- induced damage. (**A**,**B**) Protection of retinal cells by CGA. EPX ARPE-19 cells were treated with A2E, CGA (12.5 and 25 μg/mL), and blue light, as shown in Figure 2A. Cell viability was visualized using an inverted phase-contrast microscope and monitored by EZ-Cytox. (**C**) Reduction of PARP cleavage by CGA. A2E-laden RPE cells were treated with CGA (12.5 μg/mL) for 48 h before blue light illumination. Western immunoblot analysis indicated full-length PARP and cleaved PARP (cPARP) protein expression and the band intensities were quantified by densitometric analysis. The results are presented as the mean ± S.D. (*n* = 3); * *p* < 0.05. (**D**) The effect of CGA on the expression of pro-inflammatory genes. The mRNA levels were measured by RT-qPCR in CGA (25 μg/mL)-treated cells. *ATF3*—activating transcription factor 3; *XBP1s*—spliced X-box-binding protein 1; *TRIB3*—tribbles pseudokinase 3. The results are presented as the mean ± S.D. (*n* = 4); * *p* < 0.05. (**E**) The effect of CGA on A2E accumulation in ARPE-19 cells. ARPE-19 cells were pretreated with CGA (1–50 μg/mL) for 24 h before treatment with A2E-BDP (10 μM). The relative fluorescence intensity was measured using a fluorescence microplate reader. Lutein (Lu, 30 μM) was used as a positive control. (**F**) The effect of CGA on the degradation of A2E in ARPE-19 cells. Cells were incubated with A2E-BDP (10 μM) for 24 h before treatment with CGA (1–50 μg/mL). Intracellular A2E-BDP level was monitored, as shown in Figure 6. The results are presented as the mean ± S.D. (*n* = 4); * *p* < 0.05.

To investigate the active ingredient in EPX, we performed HPLC analysis. CGA, an ester of hydroxycinnamic acid, is the most abundant phenolic compound in eggplants [39]. As shown in Supplementary Figure S1, CGA was the major constituent of EPX, with a content of 17.89 mg/g (1.79%), as calculated using a standard curve. CGA exhibits prophylactic effects in vitro and in vivo against hypoxia-induced retinal degeneration [43,44] and in vivo against visible light-induced retinal degeneration [45]. Thus, we investigated whether CGA is the major active substance that inhibits the retinal damage induced by BL in A2E-laden ARPE-19 cells. First, the protective effect of CGA was measured in the A2E-BL model. Cells treated with CGA showed a significant increase in viability compared to those treated with A2E + BL (Figure 7A,B). PARP cleavage increased after A2E + BL treatment, but this phenomenon was significantly reduced after CGA treatment (Figure 7C). The upregulated expression of inflammatory genes and unfolded protein response markers (such as, CXCL8, NFKBIA, IL1B, RELA, TRIB3, and XBP1s) induced by A2E and BL was inhibited by CGA treatment (Figure 7D). Further, the intracellular A2E level was markedly reduced in the presence of CGA. However, CGA did not inhibit the accumulation of A2E in retinal cells (Figure 7E,F). Overall, these results indicate that CGA in EPX contributes to the protective effect on retinal cells against BL.

### 3.7. Effect of EPX on Blue Light-Induced Retinal Damage in BALB/c Mice

**Figure 8:**
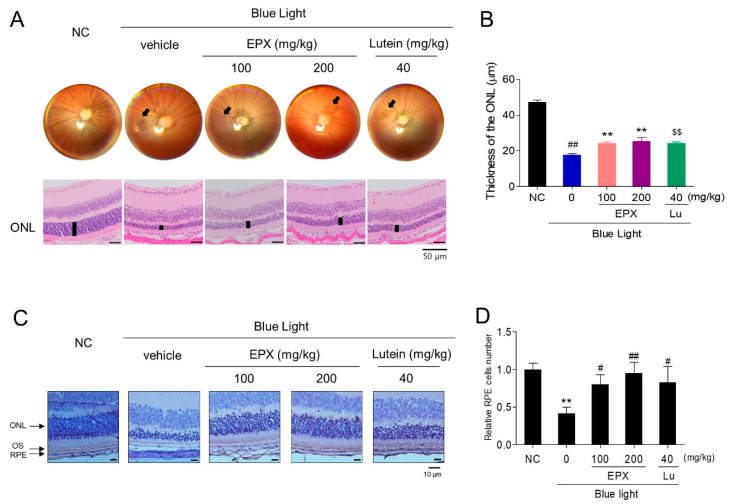
Protective effects of oral administration of EPX against retinal damage in a blue light-induced mouse model. (**A**) Histological analysis for the evaluation of morphological changes. (**B**) Measurement of the outer nuclear layer (ONL) thickness. Scale bar = 50 μm. ONL—outer nuclear layer; Lu—lutein. The results are presented as the mean ± S.D. (*n* = 8). ##—*p* < 0.01 vs. NC group (Student’s *t*-test), $$*—p* < 0.01 vs. BL group (Student’s *t*-test), **—p < 0.01 vs. BL group (One-way ANOVA). (**C**,**D**) Measurement of RPE cell number in a blue light-induced retinal damage model in mouse. ONL—outer nuclear layer, OS—outer segment, RPE—retinal pigment epithelium. The results are presented as the mean ± S.D. **—*p* < 0.01 vs. NC group (Student’s *t*-test), #—*p* < 0.05, ##—*p* < 0.01 vs. BL group (One-way ANOVA).

Next, we investigated the protective effects exerted by oral administration of EPX against retinal damage in a BL-induced retinal injury BALB/c mouse model. We observed the ocular fundus using a fundus camera after dilating the pupil; the retina was damaged in the BL treatment group compared to the control group (Figure 8A). However, we observed reduced retinal damage in EPX 100 and EPX 200 groups compared to the BL group. Based on histological analysis, we measured ONL thickness to confirm the degree of retinal degeneration. The thickness of the ONL was significantly lower in BL-treated mice than in untreated mice. Administration of EPX alleviated the degeneration of the retinal layer in a dose-dependent manner (Figure 8A,B and Supplementary Table S2), and the number of RPE cells decreased by blue light exposure was rescued by EPX (Figure 8C,D). No statistically significant weight change was observed in any of the groups during the experiment (Supplementary Figure S2). Overall, the oral administration of EPX effectively improved the retinal degeneration induced by BL illumination.

## 4. Discussion

AMD is the third leading cause of blindness in the elderly, which leads to the deterioration of the macula. Several treatment strategies have been explored for neovascular AMD, but no method has been proven effective enough to treat or prevent dry AMD. Here, we investigated the efficacy of eggplant extract for dry AMD treatment. Plant-derived antioxidants, including lutein and polyphenol compounds, have been proven to exert protective effects against A2E-induced ARPE-19 cell damage [6,46]. Recent studies have reported that *Solanum melongena* L., especially its peel, contains phenolic compounds and antioxidants Therefore, in this study, we sought to investigate the efficacy of EPX in in vitro and in vivo models of BL induced-retinal degeneration. A2E can be highly toxic to ARPE-19 cells once photo-oxidized by even at low concentrations that are normally non-toxic to RPE cells [24]. Therefore, we used the combination of A2E and BL as a suitable model to mimic dry AMD progression in vitro. Interestingly, EPX inhibited the death of RPE cells induced by A2E and BL.

Next, we tried to determine the mechanism of action of EPX. We evaluated the antioxidant efficacy of EPX. Exposure to BL induces ROS production in A2E-accumulated ARPE-19 cells, but ROS production was significantly suppressed by EPX. Therefore, EPX suppressed the apoptosis of RPE cells. Since AMD pathology involves several complex biological pathways, we used RNA-seq to investigate the in-depth mechanism of EPX action. Recent studies have shown that BL irradiation modulates various functional pathways associated with stress, immune response, apoptosis [22,34], and endoplasmic reticulum (ER) stress [47]. We analyzed the effect of BL irradiation on the differentially expressed genes and pathways, including mTOR signaling, unfolded protein response (ER stress), ROS, TNFα signaling via NF-κB pathway, inflammatory response, and p53 pathway. Notably, treatment with EPX suppressed the expression of the genes participating in most of these pathways. These results suggest that EPX acts on the upstream pathway during A2E photoactivation, as explained from its protective effect by eliminating the A2E accumulated in RPE cells.

EPX shows excellent anti-inflammatory properties. It effectively inhibited the nuclear translocation of NF-κB induced by BL irradiation in A2E-laden ARPE-19 cells. As a result, EPX downregulated the expression of pro-inflammatory target genes (*CXCL8, IL1B, ATF3, TRIB3,* and *RELA*) activated by the NF-κB pathway. The allele of *IL-8* (C-X-C chemokine ligand 8 [CXCL8]) has been shown to increase the risk of neovascular AMD [48]. Other studies have demonstrated that the destabilization of accumulated drusen or RPE lysosomes stimulates the NLRP3 inflammasome pathway, resulting in increased secretion of IL-1B and IL-18 [49,50]. We demonstrated that EPX effectively inhibited the upregulated inflammasome pathway in RPE cells after BL exposure. In addition, EPX also inhibited the NF-κB pathway induced by TNFα, suggesting that it can inhibit RPE cell death mediated by the inflammatory attack in the retina. Moreover, as A2E photoactivation induced by BL accelerates ROS production and inflammatory processes, EPX can degrade ROS in retinal cells and effectively alleviate pathological inflammation progression underlying dry AMD. Based on these results, we verified the efficacy of EPX in an animal model of retinal injury induced by BL. We analyzed the fundus and retinal layer thickness, the representative markers of retinal damage, and found that EPX significantly suppressed retinal damage induced by BL.

Another significance of this study is that CGA was identified as an active substance in EPX that may have potential application for AMD therapy. CGA, one of the most useful phenolic compounds in nature, exerts antioxidant activities; CGA exhibits free radical-scavenging and anti-inflammatory properties [51]. CGA significantly decreased the hypoxia-stimulated retinal damage in vitro and in vivo [44]. The visible light-enhanced retinal lesion in pigmented rabbits was alleviated by CGA supplementation, which repressed oxidative stress, inflammatory cytokines, and angiogenesis-associated factors [45]. However, this is the first study to report the efficacy of CGA in a dry AMD model induced by BL. We observed that CGA rescued ARPE-19 cells from BL-mediated A2E activation via inhibiting apoptosis. Further, in A2E-accumulated RPE cells, CGA attenuated pro-inflammatory gene expression (*CXCL8, IL1B,* and *TRIB3*) induced by BL illumination. CGA eliminated A2E within cells but did not inhibit A2E accumulation. This evidence clarifies the major protective effect of EPX against BL irradiation in ARPE-19 cells containing A2E. Thus, the understanding of the detailed mechanism underlying the EPX- or CGA-mediated degradation of intracellular A2E warrants further studies. The limitation of the study is the uncertainty of the active metabolite of EPX, which is the major disadvantage of oral administration compared to intravitreal injection in ophthalmology. Therefore, the identification of the active metabolites of EPX would further enhance our understanding of protective effects exerted by EPX. Notably, the metabolic pathways and metabolites of chlorogenic acid identified in this study as active ingredients of EPX, have been reported earlier.

Overall, we confirm that EPX is effective in inhibiting BL-induced retinal degeneration via removing intracellular A2E, thereby reducing ROS levels, inhibiting NF-κB signaling, and suppressing BL-induced apoptosis. In particular, it effectively suppresses the decrease in BL-induced ONL thickness in an in vivo model. In addition, CGA was identified as a key active substance in EPX. Collectively, these findings suggest the potential therapeutic or prophylactic effect of *S. melongena* L. extract against dry AMD.

## Data Availability

The data presented in this study are available on request from the corresponding author. The data are not publicly available due to confidentiality concerns.

## Supplementary Materials

The following are available online at https://www.mdpi.com/2072-6643/13/2/359/s1, Figure S1. HPLC chromatogram of chlorogenic acid from EPX, Figure S2. Changes in the body weight of mice in animal model, Table S1. Primer sequences for RT-qPCR, Table S2. Raw values of ONL thickness from BL-induced retinal degeneration model.
