# Supplementary material for: Solanum melongena L. Extract Protects Retinal Pigment Epithelial Cells from Blue Light-Induced Phototoxicity in In Vitro and In Vivo Models
Source: Nutrients. 2021 Jan 25;13(2):359. doi: 10.3390/nu13020359 (PMC7912168; doi:10.3390/nu13020359)
Supplement: Supplementary file 1 [file nutrients-13-00359-s001.pdf]

Supplementary Information

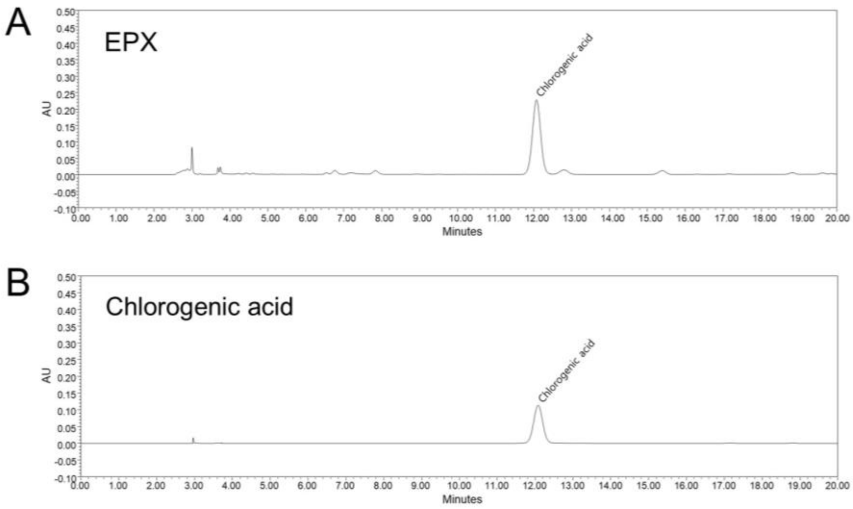

Supplementary Figure S1. HPLC chromatogram of chlorogenic acid from EPX. Chromatograms of (A) EPX and (B) chlorogenic acid as standard compound. Absorbance was measured at 310 nm wavelength.

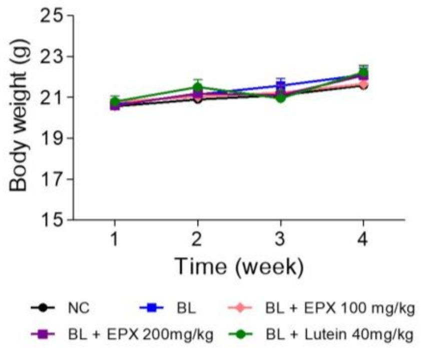

Supplementary Figure S2. Changes in the body weight of mice in animal model.

Supplementary Table S1. Primer sequences for RT-qPCR

Name	Forward	Reverse
ATF3	GCTGTCACCACGTGCAGTATCTCA	CTGTTCCTCCTCTTGCTGACAAGC
XBP1s	TGCTGAGTCCGCAGCAGGTG	GCTGGCAGGCTCTGGGGAAG
HERPUD1	CGTTGGGTGGTTTCCATTTA	TGGTTGGGGTCTTCAGTTTC
DDIT3	CTAGTGCCAATGATGTGA	ATATACAAGCTGAGACCTT
TRIB3	GAGGAGGGAGACAGAGAAG	TGGAAGGCACTGAAGGTT
HSPA5	AGCTGTAGCGTATGGTGCTG	AAGGGGACATACATCAAGCAGT
CXCL8	TGAATTACGGAATAATGAGTTAGAAC	TCAACCAGCAAGAAATTACTAAT
IL1B	CCACCTCCAGGGACAGGATA	AACACGCAGGACAGGTACAG
RELA	GTGGGGACTACGACCTGAATG	GGGGCACGATTGTCAAAGATG
NFKBIA	ACCTGGTGTCACTCCTGTTGA	CTGCTGCTGTATCCGGGTG
18s	GAGGATGAGGTGGAACGTGT	TCTTCAGTCGCTCCAGGTCT

Supplementary Table S2. Raw values of ONL thickness from BL-induced retinal degeneration model.

[ONL thickness] (μm)

GROUP: G1 (n=8) NC

Animal ID	G1-1		G1-2		G1-3		G1-4		G1-5		G1-6		G1-7		G1-8	
Left	49.50	48.11	40.30	39.36	52.94	53.54	45.41	45.94	58.23	56.23	48.86	49.41	47.83	46.57	43.82	47.26
Right	43.73	44.71	37.05	38.65	51.28	51.88	43.74	44.84	54.06	45.65	47.08	49.32	46.17	46.24	43.51	47.87

GROUP: G2 (n=8) Blue light

Animal ID	G2-1		G2-2		G2-3		G2-4		G2-5		G2-6		G2-7		G2-8	
Left	16.87	16.06	18.60	20.23	23.51	21.36	21.54	20.30	12.15	12.13	16.72	15.87	15.23	16.19	15.87	17.09
Right	15.81	16.63	20.28	26.28	22.56	21.71	22.33	22.38	12.03	10.96	14.72	15.86	16.06	17.48	13.48	13.29

GROUP: G3 (n=8) Blue light+EPX 100mg/kg

Animal ID	G3-1		G3-2		G3-3		G3-4		G3-5		G3-6		G3-7		G3-8	
Left	20.15	19.56	33.55	27.93	28.47	28.24	23.90	23.62	21.64	20.09	23.72	25.20	25.56	26.43	21.42	21.53
Right	28.35	24.95	30.87	31.86	23.25	21.08	20.76	20.36	19.41	20.33	28.47	24.69	21.27	21.57	21.96	20.95

GROUP: G4 (n=8) Blue light+EPX 200mg/kg

Animal ID	G4-1		G4-2		G4-3		G4-4		G4-5		G4-6		G4-7		G4-8	
Left	23.70	21.21	22.30	20.08	24.36	26.58	28.60	30.28	26.90	25.29	29.60	29.82	26.03	23.85	24.78	25.62
Right	25.30	24.69	20.83	23.66	24.52	25.28	27.86	26.30	26.61	25.78	23.65	22.15	23.86	25.38	25.50	25.56

GROUP: G5 (n=8) Blue light+Lutein 40mg/kg

Animal ID	G5-1		G5-2		G5-3		G5-4		G4-5		G4-6		G4-7		G4-8	
Left	28.73	31.91	24.06	24.13	23.49	23.80	25.34	23.96	22.72	21.29	18.40	19.80	16.51	18.62	26.80	25.36
Right	32.51	35.30	23.98	24.92	23.08	24.34	24.85	25.54	23.88	22.53	22.58	20.82	21.75	23.62	25.09	23.89
